# The Prevalence of Depression in Patients With Epilepsy in the Kingdom of Saudi Arabia

**DOI:** 10.7759/cureus.55570

**Published:** 2024-03-05

**Authors:** Saleh K Swailem, Fawziah A Bamogaddam, Alawi A Al-Attas

**Affiliations:** 1 Neurology and Epileptology, Department of Neurology, King Saud Medical City, Riyadh, SAU; 2 Neurology and Epileptology, National Neuroscience Institute, King Fahad Medical City, Riyadh, SAU

**Keywords:** antiseizure medications, kfmc, saudi arabia, pwe, depression

## Abstract

Objective

Among patients with epilepsy (PWE), the prevalence of depression ranges from 30% to 50%, with a 5-25% prevalence of suicide. Depression and epilepsy affect daily tasks such as driving, employment, and physical activity. Depression is the most common comorbidity among patients with epilepsy. Because both conditions involve pathophysiological changes, treating mood disorders helps treat epilepsy and vice versa. Studies about epilepsy and depression in Saudi Arabia are scarce, and no study has been conducted on this topic at King Fahad Medical City (KFMC); hence, we aimed to determine the prevalence of depression among PWE who were followed up at KFMC.

Methods

This retrospective hospital-based study was conducted at KFMC in Riyadh, Saudi Arabia. This investigation spanned a period of 10 years, from 2008 to 2018. The study included patients with PWE who were diagnosed with depression.

Results

According to a study of individuals aged 18 to 69, 73.7% of patients had been diagnosed with chronic depression (i.e., for more than a year); most of these patients had completed elementary school. Higher rates of depression were also observed among elementary school pupils, divorced women, and non-Riyadh residents. A correlation was observed between the severity of depression based on the Patient’s Health Questionnaire( PHQ-9) score, which was used to screen for depression and diabetes mellitus (DM), the number of antidepressant medications (ADM) used, the duration of antidepressant use, suicidal ideation or attempts, and the duration of depression. Epilepsy was most prevalent in the temporal lobe, accounting for 22.6% of all cases, and it was managed in 78.2% of the patients. The duration of epilepsy was significantly associated with the severity of depression.

## Introduction

Over two thousand years ago, Hippocrates elucidated a reciprocal association between depression and epilepsy. He found that individuals with a melancholic disposition typically develop epilepsy, and those with epilepsy tend to exhibit melancholic tendencies. The influencing factor for this predilection is the specific course that the illness follows [1]. Patients with PWE are more likely to suffer from depression than from any other psychiatric condition. Moreover, the lifetime prevalence estimates of depression among patients with epilepsy range from 6% to 30%, with estimates reaching 50% in more complex medical settings [2]. Despite the high prevalence of depression among patients with epilepsy, this disorder is still underdiagnosed and inadequately treated [3].

Multiple factors contribute to an increased risk of depression in patients with PWE. The most frequently investigated etiologies include genetic, neurochemical, anatomical, neurologic, and iatrogenic factors [4]. Patients and physicians have long believed that depression is a "normal" response to a chronic condition; however, this belief is no longer valid for PWE. Several studies have shown that patients with epilepsy have a greater risk of depression than those with other chronic diseases. Indeed, other studies have found that shared pathogenic mechanisms between the two diseases could account for this increased risk [5-7]. The management of mood disorders holds significant importance in the context of epilepsy treatment; conversely, both ailments exhibit similar pathological changes. In addition, it is imperative to refer individuals to a psychiatrist in instances of severe (major) depression accompanied by psychotic symptoms, active suicidal intent, or when two first-line treatment approaches have been ineffective [8]. Depression affects how well epilepsy surgery works and how well people take their anti-seizure medications (ASMs). Consequently, depression makes it more likely for newly diagnosed PWE to develop drug-resistant epilepsy (DRE) [9].

Numerous studies have been conducted in various nations; however, research on depression in Saudi patients with epilepsy is generally lacking. Furthermore, depression can be iatrogenic in PWE because of the side effects of ASMs or postsurgical treatment with DRE. Unfortunately, the treatment of depression in epilepsy patients is based on both empirical and uncontrolled study data [1]. Patients with temporal lobe epilepsy (TLE) have a higher incidence of depressive symptoms than those with extratemporal lobe epilepsy or generalized epilepsy. Indeed, interictal depressive symptoms and interictal major depressive episodes are prevalent in PWE in general but appear to be specifically related to TLE [10]. Many studies have been conducted on depression and anxiety in PWE; however, such studies in Saudi Arabia are scarce. In 2016, a study conducted in Taif, Saudi Arabia, revealed that 89% of adolescents between the ages of 12 and 18 who were diagnosed with epilepsy had indications of depression. Additionally, a recent study by Mubarki et al. found that a sizable portion (76.7%) of PWE experience symptoms of depression. Additionally, 8.7% of patients were diagnosed with severe depression, whereas 13.3% were diagnosed with moderate depression. The study revealed a significant association between depression and variables such as age, stress level, and employment position. However, depression was not significantly associated with sex, duration of drug use, or a specific type of drug used [11].

This study aimed to assess the prevalence of depression among PWE at a tertiary care hospital in Saudi Arabia, KFMC.

## Materials and methods

Study design

This retrospective hospital-based investigation was conducted at King Fahad Medical City, Riyadh, Saudi Arabia. This investigation spanned a period of 10 years, from 2008 to 2018.

Study population

The study included all adult patients diagnosed with epilepsy and found to have depression between 2008 and 2018. Individuals without epilepsy, those with incomplete medical records, those with cognitive impairments, those diagnosed with epileptic syndrome, and those experiencing psychogenic nonepileptic events were excluded from the recruitment process.

Ethical considerations and confidentiality

Only the patients’ initials were recorded, and when a patient’s name appeared on any other document, it was kept in a secure place by the investigators. The investigators maintained a personal patient identification list (patient’s initials with the corresponding patient’s names) to enable the identification of records.

The Institutional Review Board of KFMC (IRB log number 18-453) approved this study.

Concerning safety and efficacy

The study was retrospective; therefore, no evidence of harmful effects was detected.

Study interventions and procedures

Patient data were extracted from health information management (HIM) charts, with an emphasis on demographic variables, including age, sex, and medication use. Comorbidities such as diabetes, seizures, and chronic ailments were also considered. Following a diagnosis of depression, the presence or absence of ASMs determines the diagnosis. The frequency of seizures before and after treatment with ASMs and ADM was also compared. In addition, educational attainment, long-term epilepsy status, prior suicide attempts, disease controllability, employment status, and marital status were considered.

Statistical analysis

Using SPSS version 20.0 (IBM Corp, Armonk, NY, USA) qualitative and quantitative information were compared. Comparisons were made using the chi-square test and Fisher’s exact test. This study employed multivariate logistic regression analysis to examine the correlation between potential hazards and depression severity. A p-value less than 0.05 was considered to indicate statistical significance.

## Results

Our study aimed to detect the prevalence of depression in patients with epilepsy in KFMC; joint cases that were followed up in the epilepsy clinic and the psychiatric clinic were counted. A total of 3000 cases were reviewed, of which 2672 were found to have epilepsy without depression and 124 were affected by both epilepsy and depression.

The study was conducted on a wide range of age groups ranging from 18 to 69 years (mean age 34.90±10.55 years). There was a female predominance, with a female-to-male ratio of approximately 1.3:1. Sixty patients had a primary school education level (48.4%). Forty-four patients (35.5%) had a government job. Sixty-eight patients (54.8%) were married. Ninety patients (72.6%) lived outside Riyadh (Table 1).

**Table 1:** Demographic data distribution among the epilepsy and depression group (n=124).

Demographic data	No.	%
Age (years)		
18-30 years	47	37.9%
31-40 years	46	37.1%
>40 years	31	25.0%
Sex		
Male	55	44.4%
Female	69	55.6%
Level of education		
Primary school	60	48.4%
Secondary school	46	37.1%
High school	18	14.5%
Job		
Governmental	44	35.5%
Private	24	19.4%
Student	17	13.7%
Housewife	24	19.4%
Not work	15	12.1%
Marital status		
Single	41	33.1%
Married	68	54.8%
Divorced	15	12.1%
Residency		
Inside Riyadh	34	27.4%
Outside Riyadh	90	72.6%

**Figure 1:**
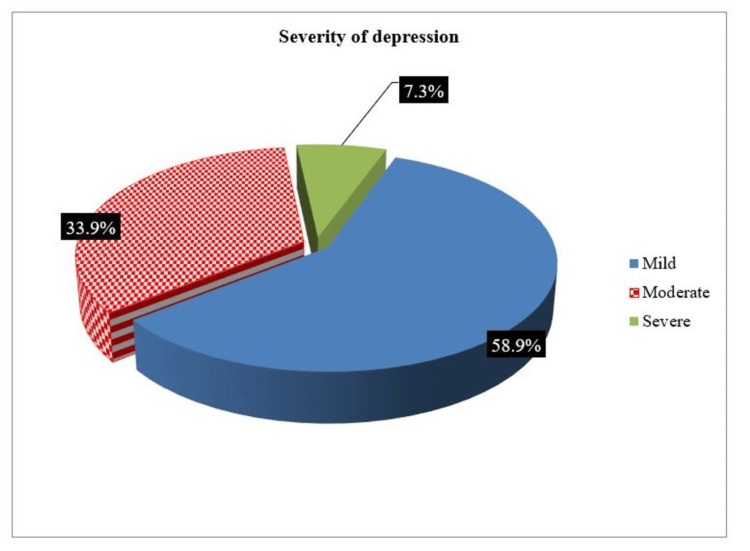
Pie chart of the severity of depression among the study group

A total of 63.7% of the patients had been diagnosed with depression for a duration exceeding one year, with the majority having previously taken antidepressants (Table 2). The majority of patients used selective serotonin reuptake inhibitors (SSRIs) for less than a year. When mild, moderate, or severe depression was present based on the PHQ-9 score, controllability was high (Table 2 and Figure 1).

**Table 2:** Depression severity and related factors among the epilepsy and depression group (n=124) ADM = antidepressant medication, SSRIs = selective serotonin reuptake inhibitors, SNRIs = serotonin and norepinephrine reuptake inhibitors, TCA = tricyclic antidepressant, NDRIs = norepinephrine and dopamine reuptake inhibitors, DM = diabetes mellitus

Depression severity & related factors	No.	%
Period of chronicity of depression		
Less than one year	45	36.3%
More than one year	79	63.7%
Number of anti-depressant medication		
No	36	29.0%
One	64	51.6%
Two	20	16.1%
>Two	4	3.2%
Type of ADM		
SSRIs	68	54.8%
SNRIs	10	8.1%
TCAs	14	11.3%
NDRIs	12	9.7%
Others	29	23.4%
Psychotherapy	8	6.5%
ADM & Psychotherapy	3	2.4%
Duration of antidepressant use		
Less than one year	65	52.4%
More than one year	59	47.6%
Controllability of depression		
Controlled	107	86.3%
Uncontrolled	17	13.7%
Severity of depression		
Mild	73	58.9%
Moderate	42	33.9%
Sever	9	7.3%
Suicidal ideation or attempt	30	24.2%
Dx of depression by whom		
Psychiatrist	81	65.3%
Epileptologist	35	28.2%
Neurologist	8	6.5%
Depression before or after ASMs start		
Before	44	35.5%
After	80	64.5%
Other commodities		
DM	9	7.3%
HTN	6	4.8%
CKD	1	0.8%
Malignancy	1	0.8%
Brain tumors	9	7.3%
Others comorbidity	112	90.3%

However, most types of epilepsy (22.6%) were temporal lobe epilepsy. Indeed, the most common age range for the onset of epilepsy was 11-20 years (33.9%). The diagnosis of epilepsy was most commonly made by epileptologists (95.2%). ASMs were most commonly taken in one dose (43.5%). The most common type of ASM was Keppra (64.5%). The most common duration of epilepsy was 11-20 years (32.3%). Most patients had controlled epilepsy (78.2%) (Table 3).

**Table 3:** Factors related to epilepsy distribution among the epilepsy and depression groups (n=124) S/P = status post, ASMs = antiseizure medications, VNS = vagal nerve stimulation

Factors related to epilepsy	No.	%
Type of epilepsy		
Generalized idiopathic epilepsy	25	20.2%
Focal epilepsy (parietal lobe, occipital lobe)	21	16.9%
Syndromic	11	8.9%
Secondary causes	9	7.3%
s/p epilepsy surgery	14	11.3%
Temporal lobe epilepsy	28	22.6%
Frontal lobe epilepsy	3	2.4%
Post-traumatic	13	10.5%
Age of onset of epilepsy		
1-5 years	21	16.9%
6-10 years	18	14.5%
11-20 years	42	33.9%
21-30 years	25	20.2%
31-40 years	11	8.9%
>40 years	7	5.6%
Diagnosis of epilepsy by whom		
Epileptologist	118	95.2%
Neurologist	6	4.8%
Number of ASMs		
Non	1	0.8%
One	54	43.5%
Two	39	31.5%
Three	22	17.7%
Four	8	6.5%
Type of ASMs		
Keppra	80	64.5%
Lamotrigine	38	30.6%
Tegretol	35	28.2%
Lacosamide	24	19.4%
Topamax	15	12.1%
Depakine	13	10.5%
Phenytoin	10	8.1%
Clonazepam	9	7.3%
Pregabalin	4	3.2%
Oxcarbazepine	3	2.4%
VNS	3	2.4%
Perambanil	0	0.0%
Duration of epilepsy by years		
>30 years	9	7.3%
1-5 years	23	18.5%
11-20 years	40	32.3%
21-30 years	17	13.7%
6-10 years	35	28.2%
Controllability of epilepsy		
Controlled	97	78.2%
Uncontrolled	27	21.8%

Furthermore, the severity of depression was significantly higher among patients with a primary school level of education, divorced patients, and patients who live outside Riyadh (p<0.001). The remaining factors were not significantly associated with the severity of depression (p > 0.05) (Table 4).

**Table 4:** Association between severity of depression and demographic data among the epilepsy and depression groups (n=124)

Demographic data	Severity of depression						Test value	P value
	Mild (n=73)		Moderate (n=42)		Sever (n=9)			
	No.	%	No.	%	No.	%		
Age group								
18-30 years	28	38.4%	13	31.0%	6	66.7%	7.291	0.121
31-40 years	29	39.7%	14	33.3%	3	33.3%		
>40 years	16	21.9%	15	35.7%	0	0.0%		
Sex								
Male	33	45.2%	17	40.5%	5	55.6%	0.735	0.693
Female	40	54.8%	25	59.5%	4	44.4%		
Level of education								
Primary school	25	34.2%	27	64.3%	8	88.9%	17.111	0.002*
Secondary school	36	49.3%	9	21.4%	1	11.1%		
High school	12	16.4%	6	14.3%	0	0.0%		
Job								
Governmental	27	37.0%	14	33.3%	3	33.3%	7.834	0.450
Private	15	20.5%	8	19.0%	1	11.1%		
Student	10	13.7%	4	9.5%	3	33.3%		
Housewife	15	20.5%	9	21.4%	0	0.0%		
Not work	6	8.2%	7	16.7%	2	22.2%		
Marital status								
Single	29	39.7%	11	26.2%	1	11.1%	35.635	<0.001**
Married	43	58.9%	23	54.8%	2	22.2%		
Divorced	1	1.4%	8	19.0%	6	66.7%		
Residency								
Inside Riyadh	28	38.4%	4	9.5%	2	22.2%	11.268	0.004*
Outside Riyadh	45	61.6%	38	90.5%	7	77.8%		

Table 5 demonstrates a statistically significant correlation between the severity of depression based on the PHQ-9 score and various factors, including the duration of depression, number of antidepressant medications, use of SNRIs, duration of antidepressant use, control of depression, suicidal ideation, suicidal attempt, the diagnosing individual, and DM (p value <0.05). The remaining factors did not have significant correlations with the degree of depression (p values >0.05).

**Table 5:** Association between the severity of depression and factors related to depression in the epilepsy and depression groups (n=124) ADM = antidepressant medication, SSRIs = selective serotonin reuptake inhibitors, SNRIs = serotonin and norepinephrine reuptake inhibitors, TCA = tricyclic antidepressant, NDRIs = norepinephrine and dopamine reuptake inhibitors

Factors related to depression	Severity of depression						Test value	P value
	Mild (n=73)		Moderate (n=42)		Severe (n=9)			
	No.	%	No.	%	No.	%		
Period of chronicity of depression								
Less than one year	34	46.6%	10	23.8%	1	11.1%	8.638	0.013*
More than one year	39	53.4%	32	76.2%	8	88.9%		
Number of anti-depressant medication								
No	30	41.1%	5	11.9%	1	11.1%	24.684	<0.001**
One	32	43.8%	28	66.7%	4	44.4%		
Two	11	15.1%	7	16.7%	2	22.2%		
>Two	0	0.0%	2	4.8%	2	22.2%		
Type of ADM								
SSRIs	34	46.6%	29	69.0%	5	55.6%	5.438	0.066
SNRIs	3	4.1%	7	16.7%	0	0.0%	6.521	0.038*
TCAs	10	13.7%	3	7.1%	1	11.1%	1.144	0.564
NDRIs	9	12.3%	1	2.4%	2	22.2%	4.766	0.092
Others	18	24.7%	8	19.0%	3	33.3%	1.004	0.605
Psychotherapy	5	6.8%	3	7.1%	0	0.0%	0.673	0.714
ADM & psychotherapy	1	1.4%	2	4.8%	0	0.0%	1.540	0.463
Duration of antidepressant use								
Less than one year	45	61.6%	19	45.2%	1	11.1%	9.516	0.009*
More than one year	28	38.4%	23	54.8%	8	88.9%		
Controllability of depression								
Controlled	68	93.2%	33	78.6%	6	66.7%	7.949	0.019*
Uncontrolled	5	6.8%	9	21.4%	3	33.3%		
Suicidal ideation or attempt	8	11.0%	16	38.1%	6	66.7%	20.250	<0.001**
Dx of depression by whom								
Psychiatrist	40	54.8%	33	78.6%	8	88.9%	9.506	0.049*
Epileptologist	26	35.6%	8	19.0%	1	11.1%		
Neurologist	7	9.6%	1	2.4%	0	0.0%		
Depression before or after ASMs start								
Before	26	35.6%	15	35.7%	3	33.3%	0.020	0.990
After	47	64.4%	27	64.3%	6	66.7%		
Other commodities								
DM	1	1.4%	7	16.7%	1	11.1%	9.482	0.009*
HTN	2	2.7%	4	9.5%	0	0.0%	3.158	0.206
CKD	1	1.4%	0	0.0%	0	0.0%	0.704	0.703
Malignancy	1	1.4%	0	0.0%	0	0.0%	0.704	0.703
Brain tumors	3	4.1%	5	11.9%	1	11.1%	2.621	0.270
Others comorbidity	69	94.5%	35	83.3%	8	88.9%	3.840	0.147

Interestingly, the severity of depression was significantly correlated with the duration and control of epilepsy. For instance, moderate depression was observed in patients with an epilepsy duration of 11-20 years (40.5%), contrary to those with severe depression (55.6%) where the duration of epilepsy was 6-10 years. Furthermore, the severity of depression was more evident in patients with uncontrolled epilepsy (22.2%). However, there was no significant correlation between the severity of depression and the type of epilepsy, age, age at onset, diagnosis, number, or type of ASMs (Table 6).

**Table 6:** Association between the severity of depression and factors related to epilepsy in the epilepsy and depression groups (n=124) GIE = generalized idiopathic epilepsy, S/P = status post, Dx = diagnosis, ASMs = antiseizure medications, VNS = vagal nerve stimulation

Factors related to epilepsy	Severity of depression						Test value	P value
	Mild (n=73)		Moderate (n=42)		Severe (n=9)			
	No.	%	No.	%	No.	%		
Type of epilepsy								
GIE	18	24.7%	7	16.7%	0	0.0%	13.947	0.454
Focal epilepsy	11	15.1%	8	19.0%	2	22.2%		
Syndromic	8	11.0%	3	7.1%	0	0.0%		
Secondary causes	5	6.8%	4	9.5%	0	0.0%		
s/p epilepsy surgery	10	13.7%	3	7.1%	1	11.1%		
Temporal lobe epilepsy	14	19.2%	11	26.2%	3	33.3%		
Frontal lobe epilepsy	1	1.4%	2	4.8%	0	0.0%		
Post-traumatic	6	8.2%	4	9.5%	3	33.3%		
Age of onset of epilepsy								
1-5 years	10	13.7%	10	23.8%	1	11.1%	10.752	0.377
6-10 years	14	19.2%	2	4.8%	2	22.2%		
11-20 years	28	38.4%	12	28.6%	2	22.2%		
21-30 years	12	16.4%	10	23.8%	3	33.3%		
31-40 years	6	8.2%	4	9.5%	1	11.1%		
>40 years	3	4.1%	4	9.5%	0	0.0%		
Dx of epilepsy by whom								
Epileptologist	68	93.2%	41	97.6%	9	100.0%	1.650	0.438
Neurologist	5	6.8%	1	2.4%	0	0.0%		
Number of ASMs								
None	1	1.4%	0	0.0%	0	0.0%	4.430	0.816
One	30	41.1%	20	47.6%	4	44.4%		
Two	26	35.6%	12	28.6%	1	11.1%		
Three	12	16.4%	7	16.7%	3	33.3%		
Four	4	5.5%	3	7.1%	1	11.1%		
Type of ASMS								
Keppra	47	64.4%	28	66.7%	5	55.6%	0.401	0.818
Tegretol	21	28.8%	12	28.6%	2	22.2%	0.173	0.917
Depakine	10	13.7%	1	2.4%	2	22.2%	5.064	0.080
Topamax	10	13.7%	3	7.1%	2	22.2%	2.013	0.365
Clonazepam	7	9.6%	1	2.4%	1	11.1%	2.272	0.321
Phenytoin	6	8.2%	4	9.5%	0	0.0%	0.912	0.634
Lacosamide	12	16.4%	10	23.8%	2	22.2%	0.979	0.613
Perambanil	0	0.0%	0	0.0%	0	0.0%	0.000	1.000
Lamotrigine	19	26.0%	16	38.1%	3	33.3%	1.860	0.395
Pregabalin	1	1.4%	2	4.8%	1	11.1%	2.916	0.233
Oxcarbazepine	1	1.4%	1	2.4%	1	11.1%	3.221	0.200
VNS	3	4.1%	0	0.0%	0	0.0%	2.148	0.342
Duration of epilepsy by years								
>30 years	7	9.6%	2	4.8%	0	0.0%	19.239	0.014*
1-5 years	18	21.9%	5	11.9%	0	22.2%		
11-20 years	22	30.1%	17	40.5%	1	11.1%		
21-30 years	12	16.4%	4	9.5%	1	11.1%		
6-10 years	14	21.9%	14	33.3%	7	55.6%		
Controlled epilepsy								
Controlled	62	84.9%	33	78.6%	2	22.2%	18.502	<0.001**
Uncontrolled	11	15.1%	9	21.4%	7	77.8%		

**Figure 2:**
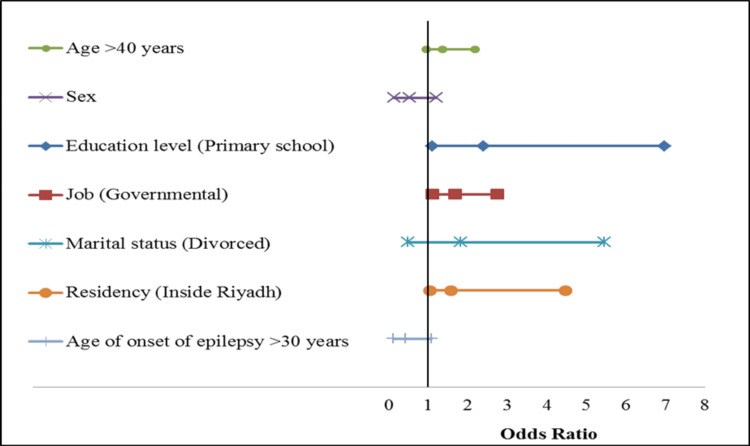
Multivariable logistic regression analysis of potential factors associated with the severity of depression

Multivariate analysis revealed that education level (primary school), marital status (divorced), and residency (inside Riyadh) were significantly associated with the severity of depression (OR (95% CI), p value) (2.392 (1.104-6.972) P=0.033; 1.822 (0.486-5.461) P=0.026; 1.574 (1.037-4.473) P=0.035, respectively) (Table 7 and Figure 2).

**Table 7:** Multivariable logistic regression analysis of potential factors associated with the severity of depression (n = 124)

Parameters	b	Wald	Sig.	OR	95% C.I.	
					Lower	Upper
Age >40 years	0.192	1.293	0.340	1.366	0.974	2.207
Sex	0.889	0.773	0.611	0.526	0.142	1.211
Education level (Primary school)	0.349	3.654	0.033*	2.392	1.104	6.972
Job (Governmental)	0.247	1.362	0.162	1.682	1.105	2.748
Marital status (Divorced)	1.549	4.353	0.026*	1.822	0.486	5.461
Residency (Inside Riyadh)	0.227	3.356	0.035*	1.574	1.037	4.473
Age of onset of epilepsy >30 years	0.548	0.711	0.677	0.435	0.128	1.080

## Discussion

This study aimed to assess the prevalence of depression among PWE at KFMC. Patients were tracked and counted in epilepsy and psychiatric clinics. A total of 3000 patients were analyzed, with 2796 having non-recurring cases, 204 having recurring cases, and 124 suffering from epilepsy and depression.

Our study revealed that 79 patients (63.7%) experienced chronic depression for more than a year, and the majority (51.6%) of the patients were prescribed a single dose of ADM. SSRIs were the most prescribed type of antidepressant, with 68 patients using these drugs for less than a year. Depression was effectively managed in 86.3% of patients. The severity of depression varied, with 58.9% mild, 33.9% moderate, and 7.3% severe. Suicidal ideation or attempts were reported in 24.2% of patients. The study also revealed a significant correlation between depression severity and the duration of epilepsy, with 78.2% of patients being effectively managed. No significant associations were found between depression severity and epilepsy type, age, or ASMs.

Similar to our study, Mubaraki et al. found that 76.7% of their cohort had depression. Although only 8.7% of the patients had severe depression, moderately severe depression was observed in 13.3% [11]. Another recent cross-sectional study conducted in Saudi Arabia found that 65.6% of participants had epilepsy, with 50.5% having a confirmed diagnosis. Depressive symptoms were prevalent in 84.7% of the participants, with a higher prevalence among younger individuals [12].

Vacca et al. studied depression severity in PWE using the Beck Depression Inventory-II. The results showed sex differences, with women reporting greater depression severity. Common symptoms included sleeping patterns, tiredness, and energy loss. Factors such as being female, having epilepsy for less than 10 years, being treated with psychotropic drugs, and reporting generalized seizures were associated with greater depression severity [13].

Seid and Mebrahtu discovered that depression was prevalent in PWE, accounting for 34.8% of cases. Depression was substantially associated with factors including an inability to read and write, prolonged medication use, a lack of improvement with ASMs, perceived stigma, and stress symptoms [14].

In newly diagnosed patients, Babtain et al. reported a significant correlation between epilepsy duration and melancholy. Patients who were diagnosed within a year and those who were diagnosed between 1 and 5 years were included in the study. The results indicate that early detection of depression in PWE is prudent, particularly in the early stages of the disease [15].

Engidaw et al. revealed that depression is prevalent among PWE in Ilu Ababore zone hospitals in Ethiopia. Factors such as lower educational status, early illness onset, poor social support, high perceived stress, high seizure frequency, and polytherapy were associated with depression. Clinicians should focus on these patients and conduct regular screenings. The study also highlighted the need for linkage with mental health service providers [16].

High perceived stigma, female sex, and seizure frequency were the factors most strongly associated with a high prevalence of depression among patients with epilepsy. According to a study by Dabilgou et al., mild, moderate, and severe symptoms of depression were reported by 67.3% of patients with epilepsy; they also found that perceived stigma, female sex, and seizure frequency were significantly associated with the prevalence of depression [17].

Research conducted by Addis et al. revealed a considerable incidence of depression among PWE, where variables including social support, perceived stigma, educational attainment, place of residence, and seizure frequency had substantial impacts. The study recommends that healthcare professionals prioritize these patients [18].

Nearly 40% of patients with epilepsy have anxiety, with thoughts being the most prevalent symptom, according to Pham et al. Anxiety rates were correlated with symptoms of depression, adverse effects of medications, tobacco use, and illicit substance abuse. In addition, anxiety results in debilitating seizures, severe epilepsy, and a diminished quality of life [19].

The impact of depression on epilepsy and seizure outcomes was investigated by Josephson et al. Treatment for depression was associated with worse outcomes, which may indicate a more severe form of depression. Acute epilepsy is associated with an increased risk of developing depression, and the type of treatment influences this risk. The relationships among sex, social deprivation status, the Charlson Comorbidity Index, and incident epilepsy were also mediated by depression [20].

Limitations of the study

This study, which was conducted at KFMC in Riyadh, aimed to evaluate the prevalence of depression among individuals with epilepsy. This was the first study conducted in Saudi Arabia, ensuring thorough data recording and comprehensive evaluation. The limitations of this study include the use of a hospital setting, a small sample size, and the lack of a multicenter cohort, which could introduce publication bias and limit the generalizability of the findings.

## Conclusions

This study sheds light on the prevalence and severity of depression in Saudi Arabia, particularly among primary school pupils, divorced women, and individuals residing outside Riyadh. This study highlights the worsening of depression in patients with chronic depression who increase their antidepressant usage over time, as well as the heightened risk of severe melancholy in individuals with epilepsy. The fact that temporal lobe epilepsy is more common and that there is no strong link between epilepsy-related factors and depression severity shows the complicated relationship between mental health and neurological conditions. The frequent prescription of SSRIs, typically for durations of less than one year, underscores the importance of tailored treatment approaches. Overall, the findings emphasize the critical importance of early diagnosis and comprehensive treatment strategies to enhance the quality of life of individuals affected by depression and epilepsy in Saudi Arabia.
